# Calcium Score Use in Isolation in Acute Chest Pain Setting - Is it Sufficient?

**DOI:** 10.5935/abc.20170116

**Published:** 2017-08

**Authors:** Tiago Augusto Magalhães, Marcio Sommer Bittencourt, Carlos Eduardo Rochitte

**Affiliations:** 1 Complexo Hospital de Clínicas da Universidade Federal do Paraná (CHC-UFPR), Curitiba, PR - Brazil; 2 Hospital do Coração - Associação Sanatório Sírio - HCor/SP, São Paulo, SP - Brazil; 3 Hospital Universitário e Instituto do Câncer do Estado de São Paulo (ICESP) - Universidade de São Paulo, São Paulo, SP - Brazil; 4 Hospital Israelita Albert Einstein e Faculdade Israelita de Ciências da Saúde Albert Einstein, São Paulo, SP - Brazil; 5 Instituto do Coração (InCor) - Hospital das Clínicas da Faculdade de Medicina da Universidade de São Paulo, São Paulo, SP - Brazil

The clinical usefulness of any test in Medicine depends on the population studied, because even an accurate test will yield no benefit if applied to the wrong population. While sensitivity and specificity are characteristics inherent in the diagnostic method, the individual probability of having a disease when the test is positive (positive predictive value, PPV) and the probability of not having the disease when the test is negative (negative predictive value, NPV) depend on the disease prevalence in the population and the individual probability of having the disease before undergoing the test, the pretest probability.

The PPV and NPV are the information that matters for clinicians. In the presence of a positive test, clinicians are interested in the patient's real probability of having the disease. However, in the presence of a negative test, Clinicians want to know the true probability of the disease even with the negative result. Thus, PPV and NPV should be considered before requesting a test, because some cases a positive test might not sufficient to confirm the presence of disease, while a negative test might not be able to exclude it safely.

The investigation of chest pain of possible cardiac origin is one of the most common examples of that duality between pretest probability of disease and sensitivity and specificity for the use of ancillary tests. The first discussion about that approach for the diagnosis of coronary artery disease (CAD) was published by Diamond and Forrester in the *New England Journal of Medicine* in 1979.^1^ The pretest probability assessment before choosing the best diagnostic test for patients with chest pain continues to be recommended in current guidelines.^2-4^

While tests coronary computed tomography angiography, exercise test and functional tests associated imaging (stress myocardial perfusion scintigraphy) are clearly indicated for chest pain investigation in different scenarios,^2,5^ the use of coronary calcium score (CCS) is not recommended to the routine assessment of chest pain in any clinical situation in Brazilian guidelines.^5^ However, some studies have suggested that CCS should be considered for individuals with chest pain and low-to-moderate pretest probability of DAC, and those studies led to CCS incorporation into the *National Institute of Health and Clinical Excellence* (NICE) guidelines in 2010.^6^ That indication was based on the use of Bayes theorem and considers the low pretest probability of the population studied and the substantial NPV of CCS. However, even the NICE recommendations, which strongly consider the cost-effectiveness of the test to choose the diagnostic method, have been recently modified. In their new version, the NICE no longer recommends CCS as part of the investigation for individuals with chest pain of possible cardiac origin, and currently recommends coronary computed tomography angiography as the first-choice test for the large majority of individuals.

Some reasons for the strong appeal of CCS use are as follows: CCS is easily performed; requires a very low radiation dose, neither stressor nor contrast agents, and no patient preparation; and has no absolute contraindication. In addition, the test has a short duration (less than 5 minutes), provides almost immediate analysis and results, and requires minimal image processing.

To extend the indication of methods originally designed or validated for a specific purpose should be carefully assessed. Even more when that extension is aimed at replacing an already established or clearly more accurate method, or avoiding its use, to achieve an important diagnostic definition. 

One reason for controversy is related to the pathophysiology of acute coronary syndrome. Patients presenting to the emergency unit with acute coronary syndrome findings have a lower load of calcified plaque and culprit lesions with predominance of the non-calcified component;^7^ therefore, to base a screening test on the presence or absence of coronary calcification to assess chest pain in the emergency room might not have a proper pathophysiological rationale. In addition, the CORE64 study, assessing symptomatic patients referred for coronary computed tomography angiography, has shown that one out of five individuals presenting to the emergency unit with acute chest pain and a CCS of zero (no coronary calcification) had significant stenosis of at least one coronary segment on invasive coronary angiography.^8^ Such data do not support any decision-making based on CCS results for patients with acute chest pain.

In the present edition of *Arquivos Brasileiros de Cardiologia*, Correia et al.^9^ have assessed the possibility of extending this controversial indication of CCS use for patients admitted to a coronary unit of a Brazilian tertiary hospital with higher pretest probability of obstructive CAD. Those authors have concluded that despite the limited PPV associated with a CCS over zero, CCS had a NPV of 90% for obstructive CAD. As expected, the CCS ability to exclude disease was higher in individuals with lower pretest probability (under 50%), whose NPV reached 95%. Finally, those authors have suggested that up to one out of four individuals would have the probability of obstructive CAD sufficiently low to allow discarding that differential diagnosis based on the presence of a CCS of zero.

The PPV and NPV estimation and Bayes theorem use in clinical practice require a tool to estimate CAD pretest probability for every patient. Despite possible problems of calibration,^10^ pretest probability scores have been well established to assess stable chest pain. However, there is no validated pretest probability score for acute chest pain. Correia et al.^9^ should be congratulated for using a local sample to derive a pretest probability score for acute CAD in patients admitted to a coronary unit. However, although the pretest probability used is adequate for the present study, it has important limitations. First, the performance of that score might be overestimated, and those results most likely do not maintain that performance when replicated to other populations. Thus, external validation of the probability score is required before extrapolating the results of the present study to clinical practice.

Although waiting for that external validation, the present study has other results that justify a deeper discussion. The authors defined that a probability of obstructive CAD lower than 10% allows discarding that diagnosis. Recommendations for stable CAD consider that probability low enough to not justify further investigation.^3,4^ However, with that approach, one out of ten patients with obstructive CAD can be discharged without the right diagnosis, which might be considered inappropriate by the team in charge of patients' care in urgency and emergency settings, such as the coronary unit.

On the other hand, if a disease probability of 10% could be considered low enough to rule out disease, 8% of the patients in this study could have been discharged without undergoing any test, because none of them would have had obstructive CAD, but one in every four patients was incorrectly classified due to a CCS over zero.

In the present study, it is worth noting the inclusion of individuals with disease probability higher than previously studied. In addition, the authors were careful enough to stratify the results according to the pretest probability and the presence or absence of alterations in resting electrocardiogram and troponin levels. For individuals with disease probability higher than 50% or for those with troponin or electrocardiographic alterations, the CCS ability to rule out disease was only reasonable (NPV of 63% and 83%, respectively). On the other hand, in those with pretest probability lower than 50%, and particularly in patients with normal electrocardiogram and troponin levels, the CCS ability to rule out disease was stronger (NPV of 95% and 100%, respectively).

Those data suggest that, in patients with normal electrocardiogram and troponin levels and a pretest probability of disease between 10% and 50%, CCS can be considered an alternative in the investigation of possible anginal acute chest pain, particularly in situations in which other assessment methods, such as coronary computed tomography angiography and functional tests, are not available or are contraindicated. However, before the routine incorporation of that strategy into clinical practice, validation and calibration of the probability score adapted to this scenario are necessary, as is the replication of the results in larger cohorts to ensure the reproducibility of the CCS ability to rule out disease in that population.

Considering the pros and cons of CCS use as a gatekeeper in chest pain assessment at the emergency unit, it is worth emphasizing the current non-adoption of CCS use in isolation, aimed at ruling out significant obstructive CAD in patients with acute chest pain, by most guidelines on cardiology worldwide.
